# O-glycosylation as a potential biomarker in Alzheimer’s disease

**DOI:** 10.17179/excli2025-8717

**Published:** 2025-10-07

**Authors:** Parteek Prasher, Mousmee Sharma

**Affiliations:** 1 Department of Chemistry, University of Petroleum & Energy Studies, Energy Acres, Dehradun 248007, India; 2 Department of Chemistry, Uttaranchal University, Dehradun 248007, India

## ⁯⁯

Besides amyloid-β and tau pathology as contemporary biomarkers for monitoring the AD progression, the clinical evidence identified the diagnostic value of post translational modifications, particularly O-glycosylation signature as an emerging tool for the detection, and as a potential treatment intervention, owing to its association with protein folding, Aβ aggregation, APP processing, signal transduction, and synaptic functioning in AD pathogenesis. Anomalous O-glycosylation fingerprints detected in the CSF and brain tissues of AD patients and animal models have been associated with the hallmark pathological features in AD, thereby presenting a promising potential as candidate biomarkers in the early-stage detection and diagnosis of the disease. The research on O-glycosylation linked AD progression is although in its infantile stage with a limited clinical and preclinical evidence, there is a pressing need for targeted investigations into O-glycosylated proteins while including multi-omics strategies for redefining their role AD diagnosis and risk stratification.

### Alzheimer's statistics

March 2023 report by the World Health Organization (WHO) presented key facts associated with dementia, where it has been highlighted that 55 million people suffer from this condition of which 60 % were reported to be the residents of low- and middle-income countries which added nearly 10 million new cases annually. The report further claimed Alzheimer's disease (AD) as the most common form of dementia contributing to 60-70 % of the above cases. Dementia was reported to be the seventh most common cause of death worldwide, which in the year 2019 alone posed significant expenditure of USD 1.3 trillion on the global economies, of which 50 % was attributed to the professional caregivers, while the remaining was credited to the unpaid informal carers, such as family members and social circle who were providing an average 5h of supervision and care per day (WHO, 2025[10]). 

A special report released by 'Alzheimer's Disease Facts and Figures' by the Alzheimer's Association, United States revealed that AD is the fifth leading cause of death in US citizens in the year 2024, and presently 6.9 million US citizens ageing 65 or above have been reported to be suffering from AD which are estimated to increase to 13.8 million by the year 2060. It has been alarming to record 119,399 deaths from AD in 2021 alone, during the COVID pandemic during which Alzheimer's was still among the top seven leading causes of death in the US. In has been interesting to note that over the past 2 decades (2000 to 2021), the annual mortality rate for heart diseases, HIV and stroke have fallen significantly, however for AD the same has witnessed a sharp increase to more than 140 %. The report further highlighted that worldwide, 11 million informal caregivers provided an estimated 18.4 million hours of care and supervision to dementia and Alzheimer's patients in the year 2023, which was valued at a cost of USD 346.6 billion, not including the emotional distress and abrupt physical and psychological health outcome of the caregivers. These expenses were found to be almost equal to the expenditure USD 360 billion incurred for long-term care and professional service for these patients in the Year 2024 (Alzheimer's Association, 2024[2]). The trends reflect a decline in the professional and community-based workforce responsible for diagnosing, treating and caring of the patients, which may be attributed to an abrupt absolute increase in the number of dementia and AD patients. Apparently, there has been a significantly higher healthcare cost for dementia and AD patients aged 65 and above and as indicated by the Medicare payments to these patients which are approximately three times higher than for those without such conditions, thereby suggesting an increased healthcare needs of these patients. The Medicaid payments for dementia and AD patients have been observed to be 22-times higher than for those without these conditions, emphasizing on the higher cost of care for these patients for a professional care and specialized services which subsequently places a considerable financial burden on the Medicare and Medicaid programs run by the government agencies. 

It was estimated that the lifetime expenses of a dementia patient were approximately USD 0.4 million, of which about 0.28 million (70 %) were borne by the untrained caregivers (family members) in the form of unpaid supervision and out of pocket expenses. Hence, the accompanying special report 'Mapping a Better Future for Dementia Care Navigation' emphasized dementia care navigator for person-centric, evidence based, culturally competent and empowered support to the patients and caregivers to ensure a sustainable, highly efficient and compassionate health care system. 

Overall, AD poses a serious physical, psychological, and financial burden with a pressing need for specialized care, early diagnosis and effective treatment interventions for which the timely identification of disease biomarkers and hallmarks is deemed to play an important role in the prognosis to prevent the onset of the disease or slowing down its progression. The heterogeneous pathobiology of AD and its frequent co-occurring with the other neurodegenerative conditions and dementias, such as Lewy Body Dementia (LBD), Frontotemporal Dementia (FTD), and Vascular Cognitive Impairment (VCI) is a major challenge in the disease prognosis, which highlights the importance of differential diagnosis for selecting personalized treatment regime. However, there occurs a significant time lag of approximately 2.8 years between the onset of symptoms and diagnosis of AD due to which several patients have been reported to have already progressed into the later stages of the disease during the diagnosis stage (Dubois et al., 2023[3]).

The contemporary treatment paradigm of AD has made significant strides owing to the existing biomarkers, including positron emission tomography (PET) imaging of β-amyloid and Tau proteins, biomarkers of cerebrospinal fluid (CSF), blood-based biomarkers and genetic biomarkers. However, their non-specificity, invasiveness, heterogeneity, and high operational cost poses concerns about the biomarker's reliability, accessibility, and utility thereby necessitating the need for novel biomarkers to improve the patient outcomes (Georgakas et al., 2023[5]). 

### Post translational modifications (PTM) 

PTMs are referred as the enzyme-guided, non-enzymatic, or covalent processing events which are responsible for altering the chemical properties of amino acids via proteolytic cleavage followed by an array of modifications, including phosphorylation, glycosylation, carbonylation, hydroxylation, galactosylation, acetylation, methylation, nitration, palmitoylation, prenylation, sulphation, ubiquitination, citrullination, carbamylation, and sumoylation that subsequently influence the structure and dynamics of the regulatory proteins (Lee et al., 2023[7]). The primary function of PTMs is to improve the proteolytic stability of proteins, and a crosstalk between the various types of PTMs is crucial for regulating the activation states of key regulatory proteins associated with cell metabolism. Ostensibly, erroneous PTMs have been reported to induce changes in the protein properties and loss in their function is subsequently implicated in diseased conditions (Zhong et al., 2023[11]). 

### O-glycosylation in AD pathogenesis

Proteolytic cleavage of amyloid β (Aβ) peptide by the β-site amyloid precursor protein cleaving enzyme 1 (BACE1) and γ-secretase produced by the transmembrane Amyloid precursor protein (APP), followed by its aggregation and deposition in the brain as amyloid plaques, along with the production of neurofibrillary tangles by phosphorylation of microtubule-associated Tau protein serve as the pathological hallmarks of Alzheimer's disease. Alteration in the *N-* and *O*-glycosylation pattern of APP, BACE1 and Tau have been reported to be implicated in the pathogenesis of AD, which makes them an ideal candidate as potential biomarkers of the disease (Haukedal and Freude, 2021[6]). 

Abnormal hyperphosphorylation of tau protein and its aggregation as neurofibrillary tangles in brain of AD patients has been reported to be guided by O-GlcNAcylation in the preclinical studies on starved mice with reduced glucose metabolism to mimic AD brain. A decreased O-GlcNAcylation was observed in these animal models, subsequently leading to tauopathy (Liu et al., 2004[8]). The evidence on the tau phosphorylation and O-GlcNAcylation as mutually exclusive post-translational modifications was provided by Gatta et al. (2016[4]) on triple transgenic (3xTg-AD) ageing mouse detected with tau protein hyperphosphorylated on both the Ser396 and Thr205 residues in frontal cortex and hippocampus. The extent of Tau O-GlcNAcylation was ascertained by studying immune-precipitates which showed a substantial reduction in the PTM in hippocampus of the test animal models, whereas no changes were observed in the frontal cortex or cerebellum. Interestingly, no changes were observed in the expression associated enzymes glutamine fructose-6-phosphate amidotransferase, O-linked β-N-acetylglucosamine transferase, and O-GlcNAc hydrolase in the hippocampus of animal models suggesting that the reduction in the PTM was not associated with changes in enzyme levels (Gatta et al., 2016[4]). 

Akasaka-Manya and coworkers explored the role of N-acetylgalactosaminyl transferases (GalNAc-Ts) mediated O-glycosylation of APP on Aβ production in the progression of AD. Real time RT-PCR analysis suggested that GalNAc-T6 considerably reduced the generation of Aβ1-40 and Aβ1-42, whereas GalNAc-T1 and GalNAc-T4 lowered the production of Aβ1-40 only, with GalNAc-T6 showing an outstanding activity against APP. Furthermore, the expression of α- and β-secretase were found to be altered in the transfected cell line although not much significant. Similarly, no difference was observed for the expression of GalNAc-T1, 2, 3, 12, 14, 15, and 16 in control and the AD patients, while the expression of GalNAc-T4, 6, 7, 8, 10 was augmented with advancing stages of AD. It was interesting to observe that GalNAc-T6 mediated excess *O*-glycosylation of APP led to a reduction in the production of Aβ, and it results in altering the susceptibility of APP towards secretases without effecting the original activity of secretases themselves. *O*-glycosylation also effects protein trafficking, their localization and conformation which can result in altering the susceptibility of proteins towards secretases. Apparently, the anomalous *O*-glycosylation changes its localization in the cells and prevents its movement to the BACE1 containing intracellular acidic compartments, including endosomes and trans-Golgi, thereby effecting Aβ generation. In elderly people with AD, a single nucleotide polymorphism (SNP) at Ala673 in APP770 and Ala598 in APP695 is reported to cause a substitution of alanine for threonine, thereby creating a potential site for *O*-glycosylation. SNP-induced alteration in APP minimizes Aβ production by 40 %, which suggests that *O*-glycosylation at this position may be responsible for enhancing TACE activity or inhibition of BACE1 activity. Excess *O*-glycosylation owing to the overexpression of GalNAc-Ts subsequently activates Src, which causes the translocation of GalNAc-Ts from Golgi to endoplasmic reticulum (ER) apparently increasing the *O*-glycosylation on APP and other proteins leading to the inhibition in the generation of Aβ (Akasaka-Manya et al., 2017[1]).

Singh et al. (2021[9]) studied alterations in the conformation and proteolytic susceptibility of mutated APP (glyco)peptides in the presence of tyrosine (Tyr^681^) *O*-GalNAc. In this study, both native and Swedish-mutated APP peptides with and without O-GalNAc modification at Tyr^681^ were synthesized. It was observed that the glycosylated peptides showed increased susceptibility towards β-secretases, which facilitated the processing of APP towards amyloidogenic pathway responsible for the formation of Aβ plaques in AD. This evidence also highlighted the importance of specific glycosylation patterns in the modulation of APP structure and its proteolytic processing which could be beneficial for the development of impending therapeutic interventions for AD management (Singh et al., 2021[9]).

### Conclusion

Alzheimer's disease poses a significant healthcare challenge and economic burden in the contemporary era. While progress has been achieved in the development of AD biomarkers, however these are largely based on amyloid and tau protein pathologies which are occasionally not able to address the multifactorial nature and clinical heterogeneity of AD. Further, AD diagnosis via these biomarkers involves invasive approaches, some of which are not widely accessible, and others are still undergoing standardization and regulatory validation for routine clinical use. Apparently, there is a pressing need for novel biomarkers for AD diagnosis to facilitate treatment interventions and personalized medicine. O-linked glycosylation has offered a promising candidature as an impending biomarker of AD owing to its regulatory role in several critical biochemical processes associated with AD pathogenesis. As such, several preclinical studies and glycoproteomic profiling assays have provided evidence on the alterations in O-glycosylation pattern of neural proteins, including APP and tau which actively contributed to AD pathology. Interestingly, most of the O-glycosylation-related modifications have been observed in the early stage of disease prior to irreversible neural damage suggesting that the O-glycosylation signature may serve as a prognostic indicator of the onset of disease. Overall, the integration of O-glycosylation fingerprints in the current biomarker landscape of AD holds promise for early diagnosis of the disease which enables patient stratification for personalized medicine and enables the elucidation of novel therapeutic targets linked with O-glycosylation mediated pathways. Future efforts need to be focused on the development of reference glycosylation signatures and correlation of glycosylation changes with the disease progression to exhaustively tap the clinical utility of O-glycosylation in AD.

## Declaration

### Conflict of interest

The authors declare no conflict of interest.

### Artificial Intelligence (AI) - Assisted Technology 

The authors declare that they have not used artificial intelligence for the writing of the manuscript.
